# Enzymatic Activities of *CYP3A4* Allelic Variants on Quinine 3-Hydroxylation *In Vitro*

**DOI:** 10.3389/fphar.2019.00591

**Published:** 2019-05-31

**Authors:** Xiao-Yang Zhou, Xiao-Xia Hu, Chen-Chen Wang, Xiang-Ran Lu, Zhe Chen, Qian Liu, Guo-Xin Hu, Jian-Ping Cai

**Affiliations:** ^1^The MOH Key Laboratory of Geriatrics, Beijing Hospital, National Center of Gerontology, Beijing, China; ^2^Department of Pharmacy, Jinhua Central Hospital, Jinhua, China; ^3^School of Pharmacy, Wenzhou Medical University, Wenzhou, China; ^4^Department of Pharmacy, Wenzhou People’s Hospital, Wenzhou, China

**Keywords:** cytochrome P450 3A4, drug metabolism, quinine, 3-hydroxylation, polymorphism

## Abstract

Cytochrome P450 3A4 (CYP3A4) enzyme activity is known to show considerable ethnic heterogeneity and inter-individual differences, affecting the outcome of drug treatment. *CYP3A4* genetic polymorphisms are believed to be one of the important causes, leading to inter-individual variability in drug metabolism. Quinine is an antipyretic drug with antimalarial properties that is metabolized primarily by CYP3A4. Quinine 3-hydroxylation has been proven as a biomarker reaction for evaluating CYP3A4 ability. Quinine has frequent adverse effects and there are distinct inter-individual differences in quinine sensitivity. The open reading frame for 30 *CYP3A4* allelic variants were constructed from wild-type CYP3A4*1A by an overlap extension polymerase chain reaction. Recombinant CYP3A4 variants were expressed using baculovirus-insect cell expression system, and their catalytic activities towards quinine hydroxylation were determined and evaluated. Of the 30 CYP3A4 allelic variants, 23 variants exhibited significantly reduced intrinsic clearance towards quinine, 2 variants showed increased intrinsic clearance for quinine, 2 variants possessed no significant differences towards quinine, compared with CYP3A4*1A, and 3 variants had no detected expression and enzyme activity. Our assessment on the enzymatic activities of CYP3A4 variants towards quinine may contribute to laying an experimental foundation for further clinical studies so as to accelerate the process of determining the associations between genetic variations and clinical phenotypes.

## Introduction

Cytochrome P450 3A4 (CYP3A4) contributes to the metabolism of ∼50% of drugs used therapeutically (Rendic, 2002; Guengerich, 2005) and also to the metabolism of endogenous substrates, such as retinoic acid, bile acids, and steroid hormones (Ingelman-Sundberg et al., 2007). It has been reported that CYP3A4 expression differs between people, with 43.4-fold differences in expression observed in human liver microsomes from different individuals (Ohtsuki et al., 2012). Further, CYP3A4 enzyme activity shows considerable ethnic heterogeneity and inter-individual differences, which have a major impact on the outcome of drug treatment (Zanger and Schwab, 2013). Inter-individual variability in CYP3A4 activity is due to environmental factors (e.g., environmental exposures, drug interactions), as well as genetic variation in the CYP3A4 gene, and results in variability in drug efficacy and adverse events (Ma and Lu, 2011; Rahmioglu et al., 2011). Evaluation of CYP3A4 activity using probe drugs is important to elucidate the impact of genetic variation on inter-individual variability in CYP3A4 activity, relative to environmental factors.

Quinine, a component of the bark of the *Cinchona* tree, has been used as an important drug for the treatment of malaria for several hundred years (Achan et al., 2011). Quinine is recommended as the treatment for uncomplicated malaria, particularly in the first trimester of pregnancy (W. H. Organization, 2015), or is used to treat severe malaria if the first-line drug fails or is not available (W. H. Organization, 2015). Further, quinine is used as a flavor modifier in beverages such as bitter lemon and tonic water (Horie et al., 2006; Bel et al., 2009), and is an ingredient of some lotions and shampoos. Quinine is eliminated over 80% *via* hepatic biotransformation and is excreted unchanged about 20% by the kidneys. Zhang et al. (1997) reported that the formation of the major metabolite of quinine, 3-hydroxyquinine, was highly correlated with CYP3A4 apoprotein levels as determined by western blotting. *In vivo* CYP3A4 has also been proven to be important for the 3-hydroxylation of quinine (Mirghani et al., 1999; Mirghani et al., 2003). Quinine has been used as a reliable probe substrate of CYP3A4 for association studies between enzymatic activities and genotypes because CYP3A4 is the major metabolic enzyme involved in quinine 3-hydroxylation (Mirghani et al., 2002; Mirghani et al., 2003; Rodriguez-Antona et al., 2005) both *in vivo* and *in vitro*.

So far, 30 *CYP3A4* variants, with amino-acid changes located in coding regions, have been identified among different ethnic population (www.pharmvar.org/gene/CYP3A4), including seven new variants, CYP3A4*28–*34, detected in our recent study (Hu et al., 2017). Along with the new allelic variants added, the demand for functional studies of CYP3A4 variants has been increasing. In view of substrate specificity for a genetic variant, our group tested various CYP3A4 substrates, including testosterone, lidocaine (Fang et al., 2017), and amiodarone (Yang et al., 2019), with these variants. In the present study, we attempted to evaluate the enzymatic activities of 31 *CYP3A4* alleles towards quinine *in vitro* using high expression of CYP3A4 holoenzymes in insect cells.

## Materials and Methods

### Chemicals and Materials

Quinine, 3-hydroxyquinine (3-OH quinine), and carbamazepine (internal standard [IS]) were purchased from Toronto Research Chemicals (Toronto, ON, Canada). Cells from the fall armyworm (*Spodoptera frugiperda* [*Sf*]21), Sf-900™ III SFM insect culture medium, fetal bovine serum, Cellfectin^®^ II Reagent, and the Bac-to-Bac™ baculovirus cell expression system were obtained from Invitrogen (Carlsbad, CA, USA). PrimeSTAR^®^ HS DNA polymerase, restriction enzymes, and a DNA ligation kit were purchased from TaKaRa Bio (Shiga, Japan). A human cDNA clone for *oxidoreductase (OR)* (catalog number SC100401) and a wild-type *CYP3A4* cDNA clone (catalog number SC125488) were obtained from Origene (Rockville, MD, USA). Mouse monoclonal anti-OR antibody, anti-CYP3A4 antibody, and anti-CYP3 antibody were from Santa Cruz Biotechnology (sc-25270, sc-53850, and sc-365415; Santa Cruz, CA, USA). Rabbit monoclonal anti-CYP3A4 antibody was purchased from Abcam (ab 3572; Cambridge, UK). A regenerating system for the reduced form of nicotinamide adenine dinucleotide phosphate (NADPH) was obtained from Promega (Madison, WI, USA). High-performance liquid chromatography-grade organic solvents and liquid chromatography–mass spectrometry-grade acetonitrile were purchased from Merck (Darmstadt, Germany). All other chemicals and reagents used were of the highest purity available.

### Construction of Recombinant Expression Vectors

The dual-expression baculovirus plasmid pFastBac™ Dual was used to express the CYP3A4 and OR proteins in *Sf*21 cells simultaneously. First, the open reading frame (ORF) of *OR* was obtained and inserted into cloning sites downstream to the P10 promoter in the baculovirus vector pFastBac Dual to create the intermediate plasmid pFastBac-OR. Second, ORF fragment of CYP3A4 *1A was isolated from wild-type *CYP3A4* cDNA (catalog number SC125488) vector using 3A4*1A-F  (5′-ACCA***GTCGAC***ATGGCTCTCATCCCAGAC-3′, introducing one SalI site) and 3A4*1A-R (5′-CCAA***ACTAGT***TCAGGCTCCACTTACGGT-3′, introducing one SpeI site). Each of the ORFs of the other *CYP3A4* variants was acquired by an overlap extension polymerase chain reaction (PCR) using wild-type *CYP3A4* ORF as the template. Then, the PCR product was digested with SalI and SpeI enzymes and ligated to the intermediate plasmid pFastBac-OR to obtain the final dual-expression baculovirus vector pFastBac-OR-CYP3A4. Sequence information regarding the site-mutation primers is shown in **Table 1**. Each of the ORF regions of the dual-expression plasmid pFastBac-OR-CYP3A4 was confirmed by sequencing.

**Table 1:** PCR primers used for the site-mutation of *CYP3A4*.

Variants	cDNA changes	Forward primer (5’–3’) [references]	Reverse primer (5’–3’) [references]
CYP3A4*1A		ACCAGTCGACATGGCTCTCATCCCAGAC	CCAAACTAGTTCAGGCTCCACTTACGGT
CYP3A4*2 (S222P)	664 T → C	CTGTTATTGGGAGAAAGAA [a, b]	TTCTTTCTCCCAATAACAG [a, b]
CYP3A4*3 (M445T)	1334 T →C	GCAAACCTCGTGCCAATGC [a, b]	GCATTGGCACGAGGTTTGC [a, b]
CYP3A4*4 (I118V)	352 A →G	CTATAGAGA**C**GGCACTTTT [a, b]	AAAAGTGCC**G**TCTCTATAG [a, b]
CYP3A4*5 (P218R)	653 C→G	AGAAAGAATCGATCCAAA [a, b]	TTTGGATCGATTCTTTCT [a, b]
CYP3A4*6 *(277 Frameshift)*	830_831 insA	TTCTGAGAGTTCAATCATC	GATGATTGAACTCTCAGAA
CYP3A4*7 (G56D)	167 G→A	ATACAAAAGTCCTTATGGT	ACCATAAGGACTTTTGTAT
CYP3A4*8 (R130Q)	389 G→A	AGCAATGATTGTAATCTCT	AGAGATTACAATCATTGCT
CYP3A4*9 (V170I)	508 G→A	TCAAGGTGATAGGCTTGCC [a, b]	GGCAAGCCTATCACCTTGA [a, b]
CYP3A4*10 (D174H)	520 G→C	CAAAGACGTGTTTCAAGGT [a, b]	ACCTTGAAACACGTCTTTG [a, b]
CYP3A4*11 (T363M)	1088 C→T	AATCTGAGCATTTCATTCA [a, b]	TGAATGAAATGCTCAGATT [a, b]
CYP3A4*12 (L373F)	1117 C→T	CCCTCTCAAATCTCATAGC	GCTATGAGATTTGAGAGGG
CYP3A4*13 (P416L)	1247 C→T	AATCTTTCAAGGAGGAACT	AGTTCCTCCTTGAAAGATT
CYP3A4*14 (L15P)	44 T→C	ACCAGTCGACATGGCTCTCATCCCAGACTTGGCCATGGAAACCTGGCTTCTCCCGGCTG [a, b]	
CYP3A4*15 (R162Q)	485 G→A	TCTGCTTCCTGCCTCAGAT [a, b]	ATCTGAGGCAGGAAGCAGA [a, b]
CYP3A4*16 (T185S)	554 C→G	GATGTGCTACTGATCACAT [a, b]	ATGTGATCAGTAGCACATC [a, b]
CYP3A4*17 (F189S)	566 T→C	TTCACTCCAGATGATGTGC [a, b]	GCACATCATCTGGAGTGAA [a, b]
CYP3A4*18 (L293P)	878 T→C	ACGAGCTCCGGATCGGACA [a, b]	TGTCCGATCCGGAGCTCGT [a, b]
CYP3A4*19 (P467S)	1399 C→T	CTTTACAAGATTTGAAGGA [a, b]	TCCTTCAAATCTTGTAAAG [a, b]
CYP3A4*20	1461_1462 insA	AACAACGGGTTTTTTCTGG	CCAGAAAAAACCCGTTGTT
*(488 Frameshift)*			
CYP3A4*21 (Y319C)	956 A→G	GCCAGTTCACACATAATGA	TCATTATGTGTGAACTGGC
CYP3A4*23 (R162W)	484 C→T	CTGCTTCCCACCTCAGATT [a, b]	AATCTGAGGTGGGAAGCAG [a, b]
CYP3A4*24 (Q200H)	600 A→T	AAAGGGGTCATGTGGATTG [a, b]	CAATCCACATGACCCCTTT [a, b]
CYP3A4*26 (R268Stop)	802 C→T	AATCCACTCAGTGCTTTTG	CAAAAGCACTGAGTGGATT
CYP3A4*28 (L22V)	64 C→G	CTGGTGCTCGTCTATCTAT [a, b]	ATAGATAGACGAGCACCAG [a, b]
CYP3A4*29 (F113I)	337 T→A	CCAGTGGGAATTATGAAAA [a, b]	TTTTCATAATTCCCACTGG [a, b]
CYP3A4*30 (R130Stop)	388 C→T	AAGAGATTATGATCATTGC [a]	GCAATGATCATAATCTCTT [a]
CYP3A4*31 (H324Q)	972 C→A	GGCCACTCAACCTGATGTC [a, b]	GACATCAGGTTGAGTGGCC [a, b]
CYP3A4*32 (I335T)	1004 T→C	AGGAGGAAACTGATGCAGT [a, b]	ACTGCATCAGTTTCCTCCT [a, b]
CYP3A4*33 (A370S)	1108 G→T	TTCCCAATTTCTATGAGAC [a, b]	GTCTCATAGAAATTGGGAA [a, b]
CYP3A4*34 (I427V)	1279 A→G	AAGGACAACGTAGATCCTT [a, b]	AAGGATCTACGTTGTCCTT [a, b]

a, Drug Des Devel Ther. 2017;11:3503–3510; b, Xenobiotica. 2019 Jan;49(1):120–126.

### Expression of the Holoenzymes of CYP3A4 Variants in *Sf*21 Cells and Microsomal Fractions

Recombinant baculoviruses expressing CYP3A4 protein and OR protein were obtained from *Sf*21 cells according to manufacturer instructions. Then, *Sf*21 cells were infected with these viruses and cultivated in Sf-900 II SFM insect culture medium containing 10% fetal bovine serum, 1× penicillin (100 U/ml)–streptomycin (0.1 mg/ml), and 4 ng/μl hemin at 27°C. After 4 days of transfection, cells were harvested and resuspended in 100 mM KPO_4_ containing 1 mM ethylenediamine tetra-acetic acid, 1 mM phenylmethane sulfonyl fluoride, and 0.25 M sucrose. After centrifugation at 1,600 × *g* for 5 min, pellets were resuspended and sonicated for 30 s on ice at 25% of its full power (750 W) using a Vibracell™ sonicator (Sonics & Materials, Newtown, CT, USA). Then, the homogenate was centrifuged at 13,000 × *g* for 20 min at 4°C to remove the precipitate. Finally, the microsomal fraction was collected by ultracentrifugation at 100,000 × g for 1 h at 4°C and the pellet resuspended in 100 mM KPO_4_ (pH = 7.4) containing 20% glycerol for storage at −80°C.

### Determination of Expression of Recombinant CYP3A4 Apoenzyme and Holoenzyme

Expression of CYP3A4 apoenzyme and OR protein was determined by Western blotting according to standard procedures. Microsomal proteins were quantified using a bicinchoninic acid protein assay kit according to manufacturer (Pierce, Rockford, IL, USA) instructions. Then, 2 μg of microsomal protein was separated by electrophoresis on 10% sodium dodecyl sulfate-polyacrylamide gels, and transferred onto polyvinylidene fluoride (PVDF) membranes. The CYP3A4 apoprotein and OR protein were detected using a rabbit anti-CYP3A4 monoclonal antibody (1:2,000 dilution) and mouse anti-OR monoclonal antibody (1:8,000 dilution) as the primary antibody, respectively. After incubation overnight at 4°C, PVDF membranes were washed four times (5 min each time). Horseradish peroxidase-conjugated goat anti-rabbit and goat anti-mouse immunoglobulin (Ig) G (1:5,000 dilution) were used as secondary antibodies. Signals were visualized with a SuperSignal™ West Pico Trial Kit (Pierce).

Levels of recombinant CYP3A4 holoenzymes were evaluated using reduced carbon monoxide difference spectroscopy (RCODS) (Omura and Sato, 1964) employing an Evolution 201 system (Thermo Scientific, Rockford, IL, USA). Microsomal preparations suspended in 0.1 M potassium phosphate (pH = 7.0) were divided equally into a sample cuvette and reference cuvette, and a baseline determined. After recording the baseline, carbon monoxide was bubbled carefully through the sample for ∼40 s in the sample cuvette. Then, a few milligrams of solid Na_2_S_2_O_4_ were used for the chemical reduction of samples in the sample cuvette. Accordingly, the spectral difference in microsomal preparations was recorded by ultraviolet–visible absorption spectrometry. The change in absorbance at 450 nm relative to that at 490 nm was converted to a concentration of cytochrome P450 by using a millimolar difference extinction coefficient of 91 (Omura and Sato, 1964; Estabrook and Werringloer, 1978).

### Assay to Measure Quinine 3-Hydroxylation

The hydroxylation of quinine by recombinant CYP3A4 variants derived from *Sf*21 cells was assayed according to a specific method. According to previous experiences (Lee et al., 1995; Dai et al., 2013; Zhou et al., 2018), we performed optimization of reaction conditions including amount of enzyme, incubation time, and reaction buffer, etc. The conditions were finally controlled and selected under the linear range of the enzymatic reaction. Briefly, the incubation mixture comprised of 100 mM phosphate buffer (pH = 7.4), purified cytochrome b5, 0.7–1.2 pmol of a recombinant CYP3A4 microsomal fraction obtained from *Sf*21 cells (CYP3A4:b5 = 1:1), and quinine (25–1000 μM). This incubation mixture was pre-incubated at 37°C for 5 min. Then, 50 µl of 4 mM NADPH was added to start the reaction in a final volume of 200 μl. The reaction was carried out at 37°C for 45 min with gentle shaking and terminated by cooling to −80°C for 15 min. Finally, the mixture was precipitated with 400 μl acetonitrile and 30 μl carbamazepine (50 μg/ml, IS). The supernatant was diluted 1:9 with water and used for subsequent measurements after centrifugation at 13,000 × *g* for 10 min at 4°C. Data are the mean ± SD of three independent experiments.

The levels of 3-OH quinine and carbamazepine (IS) were measured using ultra-high-performance liquid chromatography–tandem mass spectrometry (UPLC-MS/MS). Detection was carried out with a 1290 LC system coupled to a 6490 triple quadrupole mass spectrometer (Agilent Technologies, Santa Clara, CA, USA), with a 1.8 µm Rapid Resolution HT C18 column (3.0 × 100 mm, Agilent Technologies). The mobile phase consisted of water (A) and methanol (B). The gradient elution program was 0–2.00 min (90–10% A), 2.00–4.40 min (10% A), 4.40–4.41 min (10–90% A), and 4.41–6.00 min (90% A). The flow rate was maintained at 0.4 ml/min and the injection volume was 3 µl. The total run time was 6 min. Target compounds were detected using positive electrospray ionization with multiple reaction monitoring transitions of mass/charge ratio (*m/z*) 341.1–160.1 for 3-OH quinine and *m/z* 237.1–194.1 for carbamazepine (IS). Under the conditions mentioned above, 3-OH quinine and carbamazepine (IS) separated well and their retention times were 3.47 and 3.19 min, respectively. The capillary voltage was set to 2.0 kV in positive mode and the nebulizer pressure was set to 30 psi. The fragment voltage and collision energy were set at 380 V and 35 eV for 3-OH quinine, and 380 V and 20 eV for carbamazepine, respectively. The gas temperature was set to 200°C at a flow rate of 16 L/min.

The 3-hydroxyquinine formation rate was calculated as follows: the amount of 3-hydroxyquinine formed/incubation time/amount of CYP3A4 variant used (units: pmol/min per pmol of CYP3A4 enzyme).

### Statistical Analyses

Kinetic parameters were estimated using Prism 5 (GraphPad, San Diego, CA, USA) based on non-linear regression analyses. All values of enzyme parameters, Michaelis–Menten constant (*apparent K_m_*), maximum reaction velocity (*V_max_*), and intrinsic clearance (CL_int_, which is *V_max_/apparent K_m_*), are presented as the mean ± SD from three separate experiments. Comparisons of enzymatic activity between the wild-type and variant were performed by one-way analysis of variance using the *post hoc* Dunnett method. p < 0.05 was considered to represent statistical significance.

## Results

It is known that Baculovirus host cells are deficient in all electron-transport components required for human CYP expression (Gonzalez and Korzekwa, 1995). As shown in previous studies, coexpression of CYP3A4 and OR proteins could make increase of the catalytic activity of recombinant CYP3A4 microsome, catalytically similar to human liver microsomal CYP3A4 (Buters et al., 1994; Lee et al., 1995). In the current study, the dual-expression baculovirus vectors p-FastBac-OR-CYP3A4 were constructed. Consequently, recombinant human CYP3A4 and OR proteins were highly co-expressed in the microsomes of *Sf*21 cells to assess the enzymatic activities of CYP3A4 variants towards quinine 3-hydroxylation *in vitro*.

**Figure 1:**
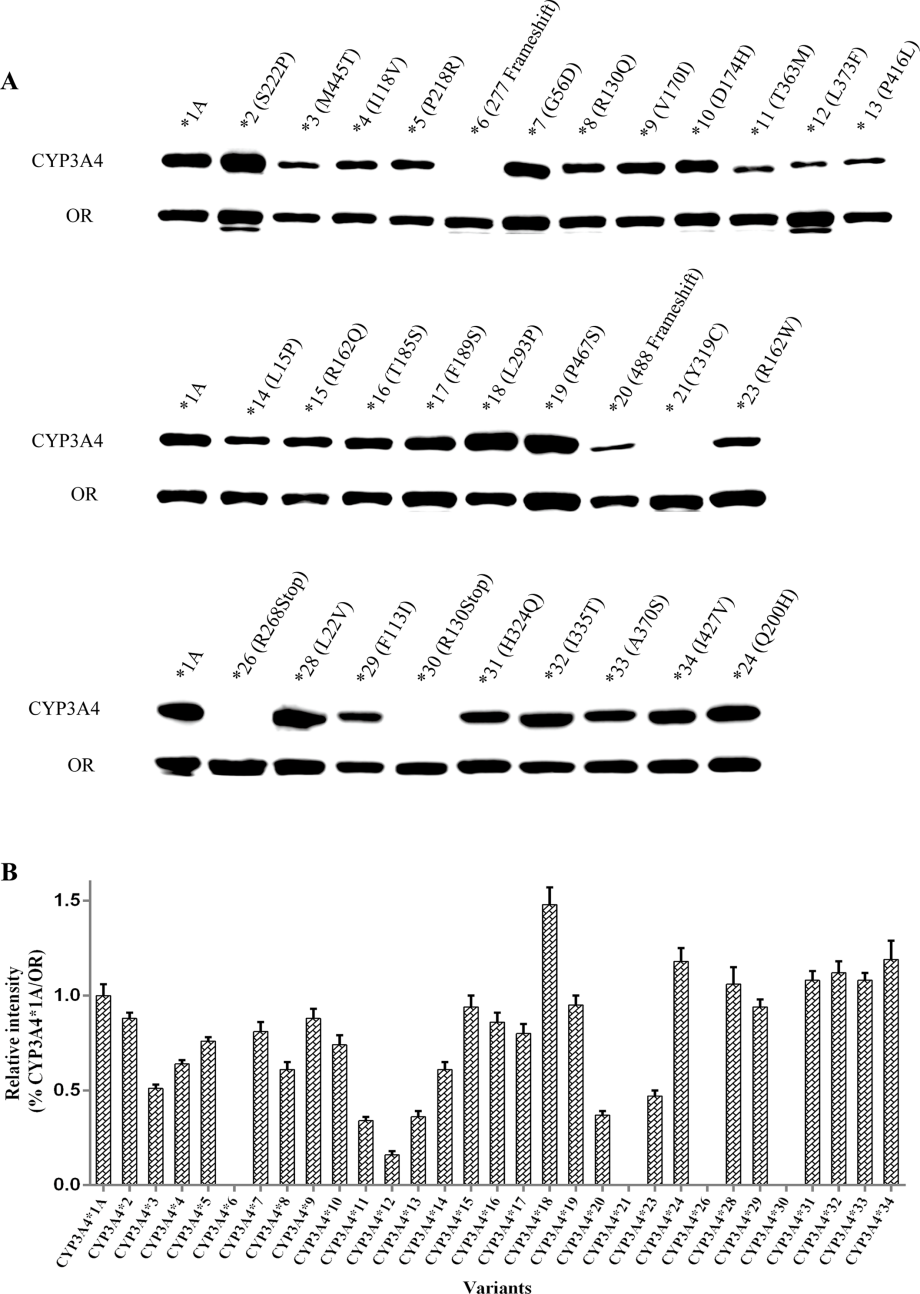
Expression of 31 *CYP3A4* alleles in *Sf*21 cells. The expressed OR and CYP3A4 proteins were detected by western blotting with antibodies against OR and CYP3A4, respectively **(A)**. Relative intensities (% of CYP3A4*1A/OR) **(B)**. Each bar represents the mean ± SD from three experiments.

Expression of recombinant CYP3A4 apoenzymes was measured by immunoblotting (**Figure 1A**). The results of the relative intensity of CYP3A4/OR indicated that CYP3A4 protein expression levels were similar across most variants (**Figure 1B**). Compared with wild-type CYP3A4*1A, several recombinant CYP3A4 variants exhibited a remarkable variation in expression. Apoprotein expression of five variants, CYP3A4*11 (T363M), CYP3A4*12 (L373F), CYP3A4*13 (P416L), CYP3A4*20 (488 frameshift), and CYP3A4*23 (R162W), was particularly lower than that of wild-type CYP3A4*1A. Conversely, one variant, CYP3A4*18 (L293P), had obviously higher expression than CYP3A4*1A. CYP3A4 apoproteins were not detected in *Sf*21 cells transfected with the allelic variants CYP3A4*6 (277 frameshift), CYP3A4*21 (Y319C), CYP3A4*26 (R268STOP), or CYP3A4*30 (R130STOP).

Recombinant CYP3A4 holoenzymes were quantified using RCODS. CYP3A4 holoenzyme was not detected for three CYP3A4 variants: CYP3A4*6 (277 frameshift), CYP3A4*26 (R268STOP), and CYP3A4*30 (R130STOP). For the other CYP3A4 variants, the concentrations of recombinant CYP3A4 holoenzymes ranged from 0.07 pmol/μl to 1.06 pmol/μl.

**Figure 2:**
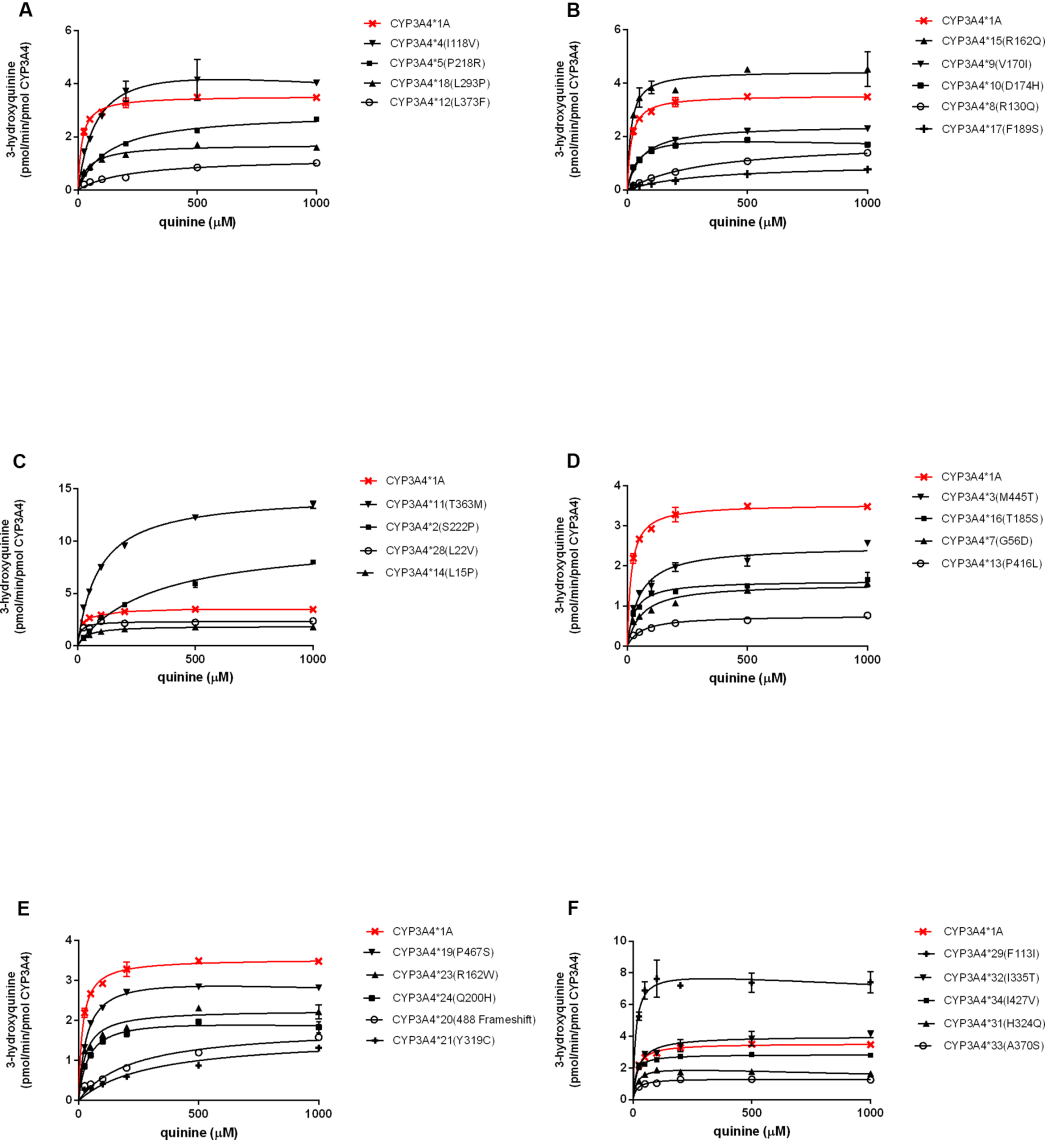
Michaelis–Menten kinetics for quinine hydroxylation by recombinant CYP3A4 variants. Each point is the mean ± SD from three experiments **(A–F)**.

The enzymatic properties of 31 CYP3A4 holoenzymes towards quinine were identified and enzymatic kinetic parameters are shown in **Table 2** and **Figure 2**. The apparent K_m_, V_max_, and CL_int_ for the 3-hydroxylation of quinine by wild-type CYP3A4*1A was 16 μM, 3.5 pmol/min per pmol CYP3A4, and 217.5 μl/min per nmol CYP3A4, respectively. Most CYP3A4 variants exhibited significantly reduced V_max_ and increased apparent K_m_. Consequently, most of the recombinant CYP3A4 holoenzymes exhibited decreased CL_int_ towards quinine, especially for the variants CYP3A4*8 (R130Q), CYP3A4*12 (L373F), CYP3A4*13 (P416L), CYP3A4*17 (F189S), CYP3A4*20 (488 frameshift), and CYP3A4*21 (Y319C), which exhibited a reduction in CL_int_ of quinine of >90% compared with that of wild-type CYP3A4*1A. However, CYP3A4*15 (R162Q), and CYP3A4*29 (F113I) proteins displayed increased CL_int_, with higher V_max_ and similar apparent K_m_, than those of wild-type CYP3A4*1A protein. In contrast, CYP3A4*28 (L22V) and CYP3A4*34 (I427V) had comparable CL_int_ to the wild-type CYP3A4 enzyme. In addition, three recombinant microsomes, CYP3A4*6 (277 frameshift), CYP3A4*26 (R268STOP), and CYP3A4*30 (R130STOP), exhibited no detectable enzymatic activity towards quinine hydroxylation, which was probably because active holoenzymes derived from these recombinant variants could not be detected (**Table 2**).

**Table 2:** Kinetic parameters for quinine hydroxylation by recombinant CYP3A4 variants.

Variant	V_max_	Apparent K_m_	Intrinsic clearance (*V_max _/apparent K_m_*)	Relative clearance^a^
	pmol/min per pmol P450	μM	μl/min per nmol P450	%
CYP3A4*1A	3.5 ± 0.1	16 ± 1.2	217.5 ± 20.6	100.0
CYP3A4*2 (S222P)	10.3 ± 0.2*	321 ± 0.8*	32.1 ± 0.4*	14.8
CYP3A4*3 (M445T)	2.5 ± 0.1*	51 ± 1.7	49.2 ± 0.8*	22.6
CYP3A4*4 (I118V)	5.7 ± 1.9*	94 ± 48.5*	64.8 ± 11.4*	29.8
CYP3A4*5 (P218R)	2.9 ± 0.1	129 ± 1.6*	22.7 ± 0.3*	10.4
CYP3A4*6 (277 frameshift)	N.D.	N.D.	N.D.	N.D.
CYP3A4*7 (G56D)	1.6 ± 0.0*	59 ± 5.9	26.6 ± 2.0*	12.2
CYP3A4*8 (R130Q)	1.8 ± 0.1*	324 ± 18.3*	5.6 ± 0.0*	2.6
CYP3A4*9 (V170I)	2.4 ± 0.1*	59 ± 1.5	40.9 ± 0.2*	18.8
CYP3A4*10 (D174H)	2.1 ± 0.1*	41 ± 4.2	51.7 ± 3.1*	23.8
CYP3A4*11 (T363M)	14.5 ± 0.5*	91 ± 8.4*	159.9 ± 9.9*	73.5
CYP3A4*12 (L373F)	1.2 ± 0.0*	216 ± 0.7*	5.6 ± 0.1*	2.6
CYP3A4*13 (P416L)	0.8 ± 0.0*	60 ± 2.3	12.6 ± 0.2*	5.8
CYP3A4*14 (L15P)	1.9 ± 0.0*	38 ± 1.1	49.7 ± 0.5*	22.9
CYP3A4*15 (R162Q)	4.5 ± 0.3	16 ± 0.7	285.2 ± 7.6*	131.1
CYP3A4*16 (T185S)	1.6 ± 0.1*	29 ± 7.3	57.5 ± 11.0*	26.4
CYP3A4*17 (F189S)	1.0 ± 0.2*	328 ± 106.1*	3.1 ± 0.4*	1.4
CYP3A4*18 (L293P)	1.7 ± 0.0*	45 ± 1.1	38.3 ± 0.2*	17.6
CYP3A4*19 (P467S)	3.2 ± 0.0	34 ± 2.6	93.5 ± 6.4*	43.0
CYP3A4*20 (488 frameshift)	1.9 ± 0.1*	227 ± 42.5*	8.3 ± 1.0*	3.8
CYP3A4*21 (Y319C)	1.6 ± 0.1*	303 ± 30.5*	5.3 ± 0.3*	2.4
CYP3A4*23 (R162W)	2.3 ± 0.1*	29 ± 0.1	77.9 ± 2.5*	35.8
CYP3A4*24 (Q200H)	2.2 ± 0.1*	43 ± 3.7	50.3 ± 1.5*	23.1
CYP3A4*26 (R268Stop)	N.D.	N.D.	N.D.	N.D.
CYP3A4*28 (L22V)	2.4 ± 0.1*	11 ± 0.7	217.7 ± 3.8	100.1
CYP3A4*29 (F113I)	8.2 ± 0.5*	12 ± 0.5	688.5 ± 73.4*	316.5
CYP3A4*30 (R130Stop)	N.D.	N.D.	N.D.	N.D.
CYP3A4*31 (H324Q)	2.1 ± 0.2*	18 ± 6.6	129.9 ± 40.0*	59.7
CYP3A4*32 (I335T)	4.0 ± 0.3	25 ± 2.1	163.5 ± 3.6*	75.2
CYP3A4*33 (A370S)	1.4 ± 0.0*	18 ± 4.4	76.2 ± 17.3*	35.0
CYP3A4*34 (I427V)	2.9 ± 0.0	12 ± 2.9	260.0 ± 64.1	119.5

Data are the means ± SD from three experiments; ND, not determined; ^a^Data are a percentage of the wild-type CYP3A4*1A; *p < 0.05 (versus wild-type CYP3A4*1A).

Paradoxically, CYP3A4*21 (Y319C) showed detectable microsomal holoenzyme by RCODS and exhibited extremely low V_max_ and high apparent Km towards quinine, with relative clearance of 2.44%; however, no detectable apoenzyme expression was found by Western blotting (**Figure 1A**, **Table 2**). We also used other commercial antibodies (sc-53850 & sc-365415, Santa Cruz, USA) to detect the expression of CYP3A4*21 but no detectable expression was identified either (results were not shown).

## Discussion

It is important to relate gene variations to clinical phenotypes using proper phenotyping tools of CYP3A4 activity. The 3-hydroxylation of quinine had been proven as a biomarker reaction for CYP3A4 activity because 3-hydroxylation of quinine is catalyzed by CYP3A4 both *in vivo* and in *in vitro* (Mirghani et al., 2003; Rahmioglu et al., 2011). Therefore, the kinetic parameters of enzymatic catalysis on quinine 3-hydroxylation were used as a measure of CYP3A4 activity in the present study.

Twenty-three of the recombinant CYP3A4 holoenzymes showed significantly decreased CL_int _towards quinine. Six variants, CYP3A4*8 (R130Q), CYP3A4*12 (L373F), CYP3A4*13 (P416L), CYP3A4*17 (F189S), CYP3A4*20 (488 frameshift), and CYP3A4*21 (Y319C), exhibited much lower CL_int_ for quinine (1.44–5.80%, **Table 2**) than wild-type CYP3A4*1A. If our *in vitro* findings are replicated in clinical studies, patients that are carriers of these alleles may be potentially CYP3A4 poor-metabolizers and may: i) require lower quinine doses to reach therapeutic plasma concentrations; and ii) be at increased risk for adverse effects and toxicity when conventional doses of quinine are used.

CYP3A4*2 (S222P) exhibited a reduced CL_int_ of more than sixfold (14.76%, **Table 2**) for quinine compared with wild-type CYP3A4*1A. This decreased enzymatic activity might be because a serine→proline amino-acid substitution could cause a major change in the three-dimensional structure of the enzyme (proline is known to break alpha-helices). Likewise, CYP3A4*2 has been reported to possess a lower CL_int_ for midazolam (Miyazaki et al., 2008), nifedipine (Sata et al., 2000; Miyazaki et al., 2008), ibrutinib (Xu et al., 2018), and lidocaine (Fang et al., 2017), than that of the wild-type enzyme. In contrast, CYP3A4*2 showed a higher CL_int_ for amiodarone (Yang et al., 2019). For testosterone, CYP3A4*2 had a decreased intrinsic clearance when expressed using *Escherichia coli* express system *in vitro* (Miyazaki et al., 2008), but the activity was comparable to the wild-type enzyme when expressed in baculovirus expression system (Sata et al., 2000) (**Table 3**).

**Table 3:** Enzyme activities of CYP3A4 allelic variants towards different substrates.

Allelic variants	Amino acid change	Enzyme activities (*in vitro*) substrates [reference]									Enzyme activities *(in vivo)* [reference] substrate
		Midazolam, (Eiselt et al., 2001; Westlind-Johnsson et al., 2006; Lee et al., 2007; Miyazaki et al., 2008; Kang et al., 2009; Maekawa et al., 2009; Maekawa et al., 2010)	Testosterone and/or chlorpyrifos, etc. (Sata et al., 2000; Dai et al., 2001; Eiselt et al., 2001; Murayama et al., 2002; Miyazaki et al., 2008; Kang et al., 2009)	Nifedipine (Sata et al., 2000; Lee et al., 2005; Miyazaki et al., 2008)	Carbamazepine, (Maekawa et al., 2009; Maekawa et al., 2010)	Atorvastatin, paclitaxel, etc. (Maekawa et al., 2010)	Lidocaine (Fang et al., 2017)	Ibrutinib (Xu et al., 2018)	Amiodarone (Yang et al., 2019)	Quinine (this study)	
CYP3A4*2	S222P	↓(Miyazaki et al., 2008)	↓(Miyazaki et al., 2008), ↔(Sata et al., 2000)	↓(Sata et al., 2000; Miyazaki et al., 2008)			↓	↓	↑	↓	
CYP3A4*3	M445T		↔ (Dai et al., 2001; Eiselt et al., 2001)	↔ (Lee et al., 2005)			↔	↑	↔	↓	
CYP3A4*4	I118V						↔	↑	↔	↓	↓(Hsieh et al., 2001) Cortisol
CYP3A4*5	P218R						↓	↓	↔	↓	↓(Hsieh et al., 2001) Cortisol
CYP3A4*6	277 Frameshift									No protein	↓(Hsieh et al., 2001) Cortisol ↓(Jun et al., 2009) Tacrolimus
CYP3A4*7	G56D	↓(Miyazaki et al., 2008)	↔ (Eiselt et al., 2001), ↓(Miyazaki et al., 2008)	↓(Miyazaki et al., 2008)						↓	
CYP3A4*8	R130Q	No protein (Eiselt et al., 2001)								↓	
CYP3A4*9	V170I		↔ (Eiselt et al., 2001)				↓	↑	↑	↓	
CYP3A4*10	D174H		↔ (Eiselt et al., 2001)				↔	↔	↑	↓	
CYP3A4*11	T363M		↓(Murayama et al., 2002), Low expression (Eiselt et al., 2001; Murayama et al., 2002)				↑	↔	↑	↓	
CYP3A4*12	L373F	↓(Eiselt et al., 2001)								↓	
CYP3A4*13	P416L	No protein (Eiselt et al., 2001)								↓	
CYP3A4*14	L15P						↑	↓	↔	↓	
CYP3A4*15	R162Q						↑	↓	↔	↑	
CYP3A4*16	T185S	↓(Miyazaki et al., 2008; Maekawa et al., 2009; Maekawa et al., 2010)	↓( Murayama et al., 2002; Miyazaki et al., 2008)	↓(Miyazaki et al., 2008)	↓(Maekawa et al., 2009; Maekawa et al., 2010)	↓(Maekawa et al., 2010)	↓	↓	↑	↓	
CYP3A4*17	F189S		↓(Dai et al., 2001)	↓(Lee et al., 2005)			Not determined	Not determined	↓	↓	
CYP3A4*18	L293P	↔ (Miyazaki et al., 2008), ↓(Lee et al., 2007; Kang et al., 2009; Maekawa et al., 2010)	↑(Dai et al., 2001; Kang et al., 2009), ↔(Murayama et al., 2002; Miyazaki et al., 2008)	↔ (Lee et al., 2005; Miyazaki et al., 2008)	↔ (Maekawa et al., 2010)	↓(Maekawa et al., 2010)	↑	↔	↑	↓	↔ (Lee et al., 2007) Midazolam ↔(Jun et al., 2009) Tacrolimus ↓ (Kang et al., 2009) Midazolam
CYP3A4*19	P467S		↔ (Dai et al., 2001)	↔ (Lee et al., 2005)			↑	↑	↑	↓	
CYP3A4*20	488 Frameshift	No activity (Westlind-Johnsson et al., 2006)								↓	↓ (Weslind-Johnsson et al., 2006) Midazolam
CYP3A4*21	Y319C									↓	
CYP3A4*23	R162W						↑	↔	↑	↓	
CYP3A4*24	Q200H						↓	Not determined	↓	↓	
CYP3A4*26	R268Stop									No protein	No protein (Werk et al., 2014), tacrolimus
CYP3A4*28	L22V						↔	↓	↔	↔	
CYP3A4*29	F113I						↑	↓	↑	↑	
CYP3A4*30	R130Stop						Not determined			No protein	
CYP3A4*31	H324Q						↑	↓	↑	↓	
CYP3A4*32	I335T						↑	↓	↑	↓	
CYP3A4*33	A370S						↔	↔	↔	↓	
CYP3A4*34	I427V						↑	↑	↑	↔	

↔, without statistical significance

CYP3A4*3 (M445T) displayed lower enzymatic activity towards quinine, compared with the wild-type allele (**Table 2**). The amino-acid residue 445 is located within the conserved heme-binding region, just two amino-acid C-terminals to the absolutely conserved Cys at position 442 in the CYP3A4 protein (Sata et al., 2000); therefore, a Met445Thr change may exert an unfavorable effect on heme binding, resulting in an alteration in catalytic activity of the enzyme, especially when binding a suitable substrate. But as noted in **Table 3**, the enzymatic activity of CYP3A4*3 was similar to that of the wild-type CYP3A4 for most substrates tested. The discrepancy of results might arise from different heterologous expression methods used, and due to differing substrate specificity for the variant.

CYP3A4*4 (I118V) exhibited lower CL_int_ towards quinine, relative to wild-type CYP3A4*1A. Studies of site-directed mutagenesis suggest that ser119 is a key amino acid implicated as an active-site residue (Yano et al., 2004). Hence, the Ile118Val mutation is likely to affect the conformation of the active site, resulting in decreased catalytic activity. Similarly, an *in vivo* study showed that the ratio of the urinary level of 6β-hydroxycortisol/free cortisol in these heterozygous individuals was lower than that in healthy controls, suggesting decreased enzymatic activities (Hsieh et al., 2001).

Both CYP3A4*8 and *13 variants displayed detectable, albeit lower, levels of CYP3A4 holoproteins and had extremely low catalytic activities towards quinine. Contrary to our results, CYP3A4*8 and *13 have been reported to exhibit no detectable CYP holoproteins when expressed in bacterial systems (Eiselt et al., 2001). We speculate that the use of bacterial vs. insect expression systems may explain the differing results. The bacterial expression system lacks an intracellular membrane environment (Gonzalez and Korzekwa, 1995) required for high expression of protein. In contrast, insect cells can process and modify proteins in a manner similar to human cells, permitting proper folding of the enzyme and incorporation of the heme cofactor, resulting in higher expression of CYP3A4 proteins.

CYP3A4*11 (T363M) was expressed at significantly lower levels than wild-type CYP3A4 (**Figure 2**), a finding that is consistent with studies using bacterial or mammalian expression systems (Eiselt et al., 2001; Murayama et al., 2002). Threonine at residue 363 within the substrate recognition site (SRS)-5 may contribute to the hydrogen bonding network formation. A substitution from threonine to methionine might result in the loss of catalytic activity (Murayama et al., 2002). So CYP3A4*11 displayed reduced quinine hydroxylation activity in current study. In addition, CYP3A4*11 showed reduced testosterone hydroxylation activity (Murayama et al., 2002), had remarkably increased CLint towards lidocaine (Fang et al., 2017) and amiodarone (Yang et al., 2019), but was not associated with noticeable changes in metabolic activities for ibrutinib (Xu et al., 2018) (**Table 3**).

CYP3A4*12 (L373F) displayed a dramatically decreased CL_int_ towards quinine, similarly with a previous research result towards midazolam (Eiselt et al., 2001). CYP3A4*12 possesses an amino-acid change of Leu373Phe; the mutated residue is located near Arg-372 and Glu-374, which participate in formation of a hydrogen-bonding network and are involved in active sites according to studies involving site-directed mutagenesis (Yano et al., 2004). Hence, the mutational residue (L373F) is likely to affect the stability of the hydrogen-bonding network and spatial conformation of the active-site cavity, resulting in altered catalytic activity of CYP3A4*12.

CYP3A4*17 (F189S) was found in Caucasians (Dai et al., 2001), Moroccans (Fernandez-Santander et al., 2016), Libyans (Fernandez-Santander et al., 2016), and Chinese (Du et al., 2006) with a frequency of 2.1%, 1.8%, 1%, and 2%, respectively. CYP3A4*17 displayed an especially low CL_int_ towards quinine compared with the wild-type. Similarly, it also exhibited extremely low catalytic activities towards testosterone (Dai et al., 2001), chlorpyrifos (Dai et al., 2001), amiodarone (Yang et al., 2019), and nifedipine (Lee et al., 2005) (**Table 3**).

CYP3A4*18 (L293P) exhibited decreased CL_int_ towards quinine (17.62%, **Table 2**). The Leu293Pro substitution has an influence on the overall protein structure and leads to the modification of the arrangement of SRS regions, the important sites for substrate recognition and substrate access to the active site (Kang et al., 2009). As described in *in vitro* studies, CYP3A4*18 displayed decreased metabolic activities towards midazolam (Lee et al., 2007; Kang et al., 2009; Maekawa et al., 2010), paclitaxel (Maekawa et al., 2010), docetaxel (Maekawa et al., 2010), and irinotecan (Maekawa et al., 2010), had increased catalytic activities towards lidocaine (Fang et al., 2017), amiodarone (Yang et al., 2019), testosterone (Dai et al., 2001; Kang et al., 2009), chlorpyrifos (Dai et al., 2001), and estrogens (Kang et al., 2009), but exhibited unchanged relative activities for nifedipine (Lee et al., 2005; Miyazaki et al., 2008), carbamazepine (Maekawa et al., 2010), and ibrutinib (Xu et al., 2018). There were also conflicting results when regarding midazolam (Miyazaki et al., 2008) or testosterone (Murayama et al., 2002; Miyazaki et al., 2008) as substrates (**Table 3**). An *in vivo* study indicated that CYP3A4*1*18-heterozygous subjects did not appear to have a significant change in midazolam clearance compared with CYP3A4*1*1 wild-type subjects (Lee et al., 2007). However, another *in vivo* study indicated that CYP3A4*18 carriers, including 52 of heterozygotes and a homozygote, showed diminished midazolam clearance (Kang et al., 2009), which is consistent with the results obtained from *in vitro* experiments (Lee et al., 2007; Kang et al., 2009; Maekawa et al., 2010).

CYP3A4*20 (488 frameshift) showed remarkably diminished apoprotein expression (**Figure 1**) and dramatically reduced CL_int_ towards quinine. In contrast, no catalytic activities of midazolam 1′- and 4-hydroxylation have been reported in yeast or HEK 293 microsomes expressing CYP3A4*20 (Westlind-Johnsson et al., 2006). These conflicting results may be attributable to the different heterologous expression systems and substrates used in the experiments. In addition, a CYP3A4*20 heterozygote, identified in Brazilian individuals, has been reported to exhibit reduced systemic clearance of ∼1.9-fold for midazolam *in vivo* (Westlind-Johnsson et al., 2006). It was hypothesized previously that this rare mutation could influence protein folding, heme incorporation (Westlind-Johnsson et al., 2006), and the spatial conformation of active-site cavity, giving rise to a decrease or loss of catalytic activity associated with the substrate used.

CYP3A4*21 (Y319C), which was identified in Chinese population (Zhou et al., 2011), displayed weak catalytic activity towards quinine in our study. Interestingly, no detectable apoenzyme expression was identified in our results. Tyr319 is a highly conserved residue in the cytochrome P450 family throughout eukaryote evolution from nematode to human (Zhou et al., 2011). According to *in silico* functional predictions (Zhou et al., 2011), Tyr319 residue lies on an important domain and a substitution of Tyr with Cys at position 319 of the CYP3A4*21 protein may induce dramatic alterations in active-site cavity configuration. Therefore, the CYP3A4*21 may produce a malfunctional protein with reduced catalytic activity towards quinine. Additionally, we speculated that these antibodies we used to detect CYP3A4 recombinant apoproteins may not be suitable to detect the variant CYP3A4*21. That is, the mutation of Tyr319Cys in CYP3A4*21 may prevent these antibodies from recognizing the specific epitopes in CYP3A4.

In contrast, two of the recombinant CYP3A4 holoenzymes, CYP3A4*15 (R162Q) and CYP3A4*29 (F113I), displayed significantly increased CL_int _for quinine. Similarly, both CYP3A4*15 and CYP3A4*29 exhibited increased CL_int_ towards lidocaine (Fang et al., 2017), but showed decreased CL_int_ for ibrutinib (Xu et al., 2018). CYP3A4*15 was detected first in different ethnic populations with small samples for each ethnic group (Lamba et al., 2002). CYP3A4*29 was identified and named in our recent study (Hu et al., 2017). The two variants are related to rapid metabolism of quinine *in vitro*. If confirmation of increased quinine clearance is found in individuals who are homozygous or heterozygous carriers of CYP3A4*29 or CYP3A4*15, these individuals may be CYP3A4 ultra-rapid metabolizers of quinine. Patients with these genotypes may: i) have poorer therapeutic efficacy when usual clinical doses of quinine are administered; and ii) require higher doses to reach therapeutic plasma concentrations.

No active holoenzymes of the CYP3A4*6 (277 frameshift), CYP3A4*26 (R268Stop), and CYP3A4*30 (R130Stop) variants were detected and, accordingly, they exhibited no metabolic activity upon quinine (**Table 2**).

CYP3A4*6 has an insertion of an adenine residue in exon 9 (830–831 insA); this mutation causes a frameshift and leads to the translation of a truncated protein that cannot incorporate heme (Hsieh et al., 2001). Consequently, the catalytic activity of CYP3A4*6 towards quinine was not identified in our study. As shown previously *in vivo*, a Chinese person was found to be heterozygous for CYP3A4*6 with a much lower ratio of urinary level of 6β-hydroxycortisol:free cortisol than wild-type individuals, suggesting decreased CYP3A4 activity (Hsieh et al., 2001). Similarly, a patient who is heterozygous for CYP3A4*6, undergoing organ transplantation, had a tacrolimus concentration-to-adjusted dose ratio 4.3-fold higher than that of wild-type patients (Jun et al., 2009).

CYP3A4*26 has a C→T nucleotide substitution in exon 5. This causes a change from arginine to a premature stop codon at position 268, and results in a putative shortened protein lacking catalytic domain and therefore activity (Werk et al., 2014). CYP3A4*26 may not be expressed due to the premature stop codon at position 268 or if it is, the protein may be marked for rapid degradation (Werk et al., 2014). Accordingly, we were unable to detect the holoprotein of the CYP3A4*26 variant expressed in *Sf*21 cells. Likewise, a 19-year-old kidney-transplanted patient, identified as a homozygote for CYP3A4*26 and meanwhile also a homozygote for CYP3A5*3, exhibited an unexpectedly high plasma level of tacrolimus as a result of extremely diminished tacrolimus clearance (Werk et al., 2014).

Similar to CYP3A4*26, CYP3A4*30 has a change from arginine to a premature stop codon at position 130 (Hu et al., 2017). The Arg130 is considered important for heme incorporation (Eiselt et al., 2001) and thus mutation of Arg130 would disrupt heme incorporation and so affect holoenzyme expression of CYP3A4.

In combination with previous studies (**Table 3**), some *CYP3A4* allelic variants showed the different preferences for the substrates unlike those of the wild type enzymes. Namely, some variants exhibit altered catalytic activities with a substrate-dependent profile. As discussed above, conflicting results deriving from a specific substrate in studies may arise from many factors such as discrepancies from *in vitro* or *in vivo* method and different heterologous expression systems.

Quinine has frequent adverse effects, because of its narrow therapeutic index (Bateman and Dyson, 1986). These adverse reactions were not only induced by drugs; they were also caused by common quinine-containing beverages. Based on a systematic review on clinical data from one hundred-fourteen articles, adverse reactions, which were resulted from quinine-containing beverages, accounted for 20% of the total adverse reactions to quinine (Liles et al., 2016). Surprisingly, even with minute exposure from common beverages, some individuals experienced severe adverse reactions involving multiple organ systems. Given obvious inter-individual diversity in quinine sensitivity, variation in CYP3A4 may be one possible mechanism underlying quinine sensitive and adverse effects. In addition, regulations for quinine use have not been established in some countries, such as, pills containing 50 mg or less of quinine are regarded as a natural health product and are available without a prescription in Canada (Liles et al., 2016); quinine-containing lotions and shampoos, as well as quinine-containing beverages, remain commonly available and supervision is not required. Thus, increased awareness of inter-individual differences in quinine sensitivity and adverse reactions to quinine among physicians and the public is believed to be necessary.

## Conclusions

In conclusion, 31 *CYP3A4* alleles were expressed using the baculovirus insect cell expression system and their catalytic abilities towards quinine were evaluated. Most recombinant CYP3A4 variants exhibited significantly decreased intrinsic clearance towards quinine. To our knowledge, this is the first study to describe functional activities of all reported CYP3A4 variants with amino acid substitutions, in quinine 3-hydroxylation. Our results may provide one possible mechanism underlying the inter-individual differences in quinine sensitivity. Further clinical studies are needed to confirm whether these CYP3A4 variants mentioned above are relevant to the variability in quinine metabolism *in vivo*. The study may contribute to providing an experimental basis for further clinical studies on quinine and so accelerate the process for establishing a genotype-phenotype connection.

## Data Availability Statement

The raw data supporting the conclusions of this manuscript will be made available by the authors, without undue reservation, to any qualified researcher.

## Author Contributions

All authors contributed to this work and agreed to submit the final version. X-YZ, G-XH, and J-PC organized and designed the study. X-YZ, X-XH, C-CW, X-RL, ZC, and QL conducted the experiments. X-YZ performed data analysis and wrote the draft of the manuscript. G-XH and J-PC contributed to the final version of the manuscript.

## Funding

This work was supported by a grant from the Ministry of Science and Technology of the People’s Republic of China (2017ZX09304026).

## Conflict of Interest Statement

The authors declare that the research was conducted in the absence of any commercial or financial relationships that could be construed as a potential conflict of interest.

## Abbreviations

CYP3A4, Cytochrome P450 3A4; RCODS, reduced carbon monoxide difference spectroscopy; URM, ultra-rapid-metabolizer; IS, internal standard; UPLC-MS/MS, ultra-high-performance liquid chromatography–tandem mass spectrometry.
